# Oncogenic lncRNA BBOX1-AS1 promotes PHF8-mediated autophagy and elicits sorafenib resistance in hepatocellular carcinoma

**DOI:** 10.1016/j.omto.2022.12.005

**Published:** 2022-12-23

**Authors:** Haisu Tao, Yuxin Zhang, Jiang Li, Junjie Liu, Tong Yuan, Wenqiang Wang, Huifang Liang, Erlei Zhang, Zhiyong Huang

**Affiliations:** 1 Department of Hepatobiliary Surgery, Zhujiang Hospital, Southern Medical University, Guangzhou, China; 2 Hepatic Surgery Center, Tongji Medical College, Tongji Hospital, Huazhong University of Science and Technology, Wuhan, China; 3 Hubei Key Laboratory of Hepato-Pancreato-Biliary Diseases, Wuhan, China; 4 The First Affiliated Hospital, College of Medicine, Shihezi University, Shihezi, Xinjiang, China; 5 NHC Key Laboratory of Prevention and Treatment of Central Asia High Incidence Diseases, Beijing, China

**Keywords:** hepatocellular carcinoma, BBOX1-AS1, tumor progression, autophagy, sorafenib resistance, PHF8

## Abstract

Some long non-coding RNAs (lncRNAs) have been documented to be involved in cancer progression and anticancer drug resistance in hepatocellular carcinoma (HCC). Thus, approaches designed to target these genes may facilitate the development of promising strategies for treating HCC. Previously, we showed that lncRNA BBOX1-AS1 was highly expressed and played an oncogenic role in HCC. However, the potential functions and mechanisms through which BBOX1-AS1 regulates HCC progression and drug resistance remain unclear. This study revealed that BBOX1-AS1 could promote tumor progression, autophagy, and drug resistance by upregulating PHF8 in HCC cells. Mechanistically, BBOX1-AS1 enhanced the stability of PHF8 mRNA by targeting the PHF8 inhibitor miR-361-3p to regulate tumor progression and autophagy in HCC. The functional rescue experiments showed that PHF8 acted as a key factor in regulating the biological effects induced by BBOX1-AS1 and miR-361-3p in HCC, indicating that BBOX1-AS1 promotes tumor progression and sorafenib resistance by regulating miR-361-3p/PHF8. Finally, mouse tumor models and patient-derived organoid models were established to further confirm these findings. Taken together, the results demonstrate that BBOX1-AS1 promotes HCC progression and sorafenib resistance via the miR-361-3p/PHF8 axis.

## Graphical abstract

**Figure:**
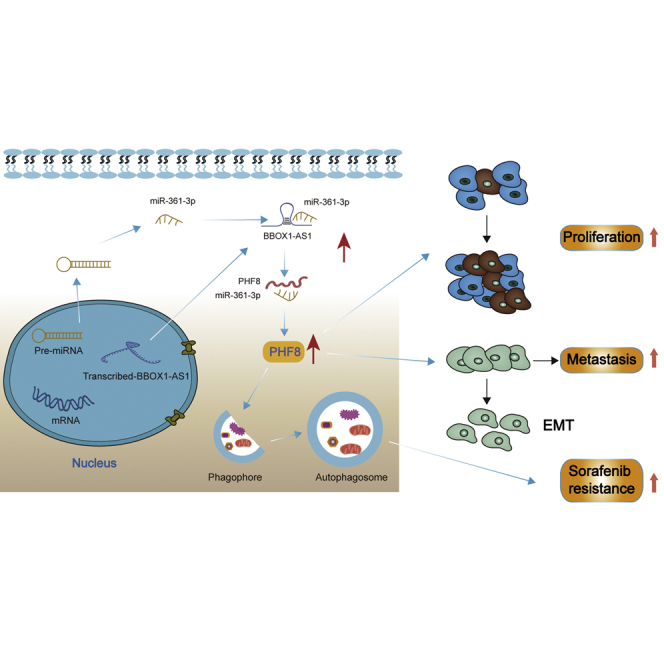

## Teaser

Some lncRNAs can be involved in progression and anticancer drug resistance in HCC. Thus, approaches designed to target these genes may become promising strategies for treating HCC. In this study, we for the revealed that BBOX1-AS1 could promote tumor progression, autophagy, and drug resistance by upregulating PHF8 in HCC.

## Introduction

Primary liver cancer is one of the deadliest health burdens in the world, and HCC is the main type, accounting for 75%–85% of liver cancer cases.^1^^,^^2^ Although 72% of new HCC cases occur in Asia, the incidence rate in Western countries has been increasing in recent years.^3^^,^^4^ There is a lack of effective early diagnostic methods, and few treatment options are available for advanced HCC patients, leading to poor prognosis for these patients.^1^ Sorafenib, which acts by inhibiting cell proliferation and angiogenesis, has become a globally recognized first-line chemotherapeutic agent and the standard therapy for inoperable advanced HCC.^5^^,^^6^ However, although sorafenib offers survival benefits for advanced HCC patients and is cost-effective, its efficacy is significantly limited by the development of drug resistance.^7^ Accordingly, sorafenib resistance is still a major obstacle affecting the treatment of HCC patients.^8^ As a result, further studies are needed to explore the underlying molecular mechanism of sorafenib resistance and to seek novel molecular targets.

Non-coding RNAs (ncRNAs), which mainly include lncRNAs, circRNAs, and miRNAs, are widely involved in the regulation of numerous oncogenic processes, including cancer progression and anticancer drug resistance.^9^ For instance, it has been shown that SNHG1 facilitates the cancer progression of HCC by upregulating miR-376a as well as downregulating FOXK1 and Snail.^10^ SNHG1 also promotes sorafenib resistance by upregulating SLC3A2 and activating the Akt pathway.^11^ Furthermore, miR-361-3p was reported to be involved in the propagation of tumor-initiating cells, TACE（transcatheter arterial chemoembolization, and sorafenib response in HCC.^12^ Therefore, approaches targeting these ncRNAs that promote tumor progression and drug resistance represent a promising strategy to improve the survival of HCC patients. Nevertheless, the molecular mechanisms through which ncRNAs exert their roles in HCC remain poorly understood and thus require further research.

In a previous study, we showed that BBOX1-AS1 was highly expressed in HCC and closely related to vascular invasion and poor prognosis.^13^ In the following study, we found a positive correlation between BBOX1-AS1 expression and the expression level of plant homeodomain finger-containing protein 8 (PHF8), a key trigger of autophagy initiation and EMT (epithelial-mesenchymal transition) in HCC. By further exploration, we found that BBOX1-AS1 was also involved in autophagy regulation and could regulate sorafenib sensitivity by activating autophagy in HCC. Mechanistic studies indicated that BBOX1-AS1 might function as a competing endogenous RNA (ceRNA) that regulates miR-361-3p and thus affects the expression level of PHF8. Taken together, our results reveal that BBOX1-AS1 promotes tumor progression and sorafenib resistance by regulating miR-361-3p/PHF8, indicating new therapeutic strategies for the treatment of HCC.

## Results

### BBOX1-AS1 is upregulated in HCC tissue and its high expression is associated with a poor prognosis

**Figure 1:**
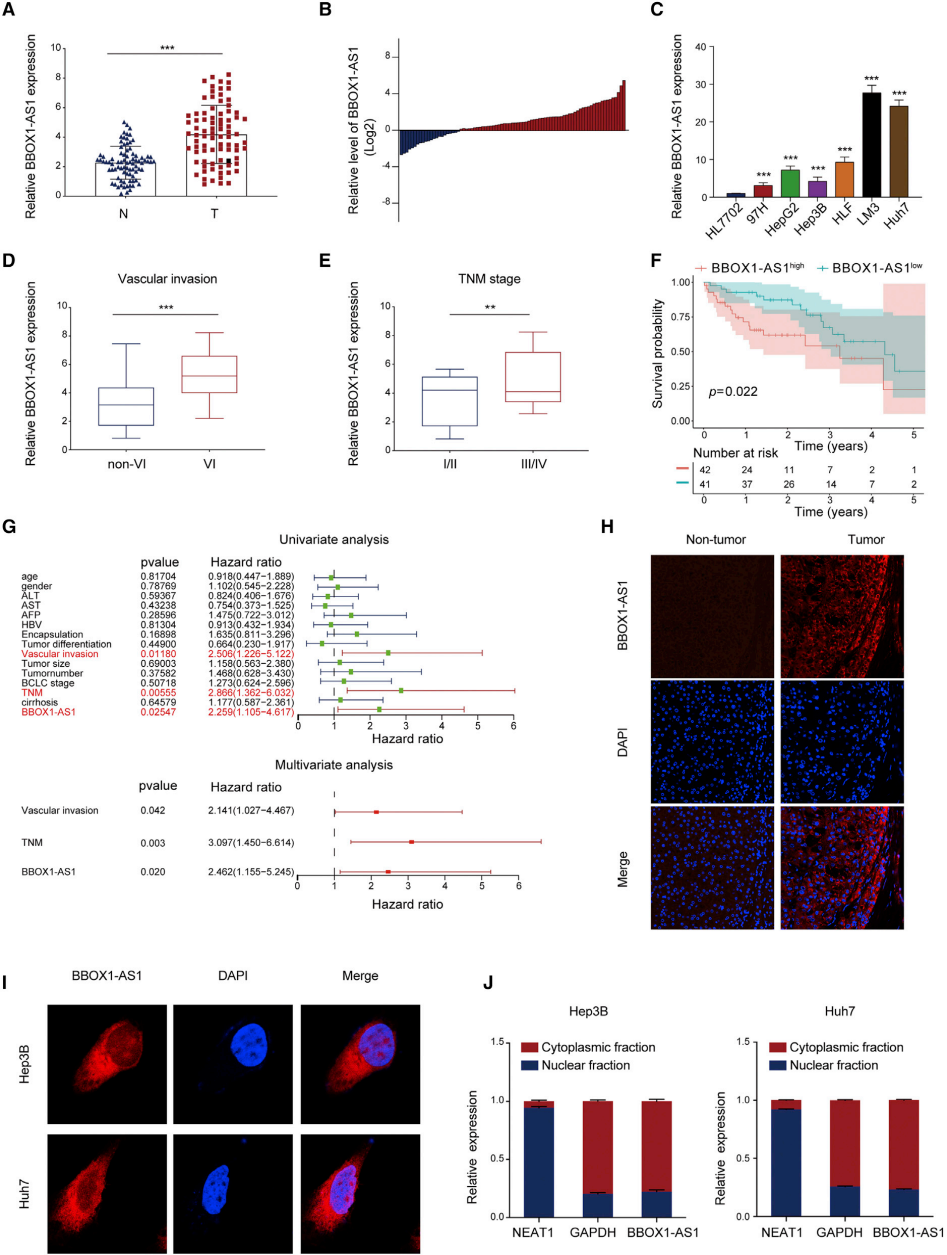
BBOX1-AS1 is upregulated in HCC tissues (A) qRT-PCR analysis of BBOX1-AS1 expression in HCC tissues and matched adjacent normal tissues (n = 83). (B) The relative expression of BBOX1-AS1 in 83 paired tissues [log2(T/N)]. (C) BBOX1-AS1 expression in normal human liver cell line HL7702 and six HCC cell lines (97H, HepG2, Hep3B, HLF, LM3 and Huh7). (D) BBOX1-AS1 expressions were compared between patients without microvascular invasion (MVI) and those with MVI (n = 83). (E) BBOX1-AS1 expression in the TNM stage I/II patients and the TNM III/IV patients (n = 83). (F) Kaplan-Meier survival curve was used to explore the effect of BBOX1-AS1 on overall survival (n = 83). (G) Cox proportional hazards analysis of the independent predictive factors for overall survival (n = 83). (H) FISH was used to explore BBOX1-AS1 expression in HCC tissues and matched adjacent normal tissues. (I) FISH was used to explore BBOX1-AS1 expression in HCC cells. (J) The expression level of BBOX1-AS1 in the subcellular fractions of HCC cells was detected by qRT-PCR. ∗∗p < 0.01, ∗∗∗p < 0.001; N, normal tissue; T, tumor tissue.

BBOX1-AS1 is highly expressed in various tumors and can promote their proliferation and metastasis.^14^^,^^15^ Our previous study also observed that BBOX1-AS1 was upregulated in HCC tissues, and its high expression was associated with poor clinicopathological characteristics and prognosis in TCGA database.^13^ To verify this finding, we determined the expression levels of BBOX1-AS1 in 83 pairs of tumor and adjacent normal tissue samples from the Tongji cohort. The results indicated that BBOX1-AS1 was markedly overexpressed in HCC tissues (Figures 1A and 1B). Additionally, BBOX1-AS1 expression was higher in HCC cells than in normal human hepatocyte cell line HL7702 (Figure 1C). Consistent with the results from TCGA database, a significant increase of BBOX1-AS1 expression was detected in HCC patients with presence of vascular invasion and advanced TNM stage (Figures 1D and 1E). Moreover, Kaplan-Meier curve revealed that higher BBOX1-AS1 expression predicted poorer overall survival (Figure 1F). A Cox proportional hazards model showed that high BBOX1-AS1 expression, vascular invasion, and advanced TNM stage were independent risk factors for worse overall survival (Figure 1G). FISH (fluorescence *in situ* hybridization) staining verified a markedly higher expression of BBOX1-AS1 in HCC tissues compared with adjacent non-tumor tissues (Figure 1H).

Taken together, the results showed that BBOX1-AS1 is upregulated in HCC, and this upregulation is associated with a poor prognosis.

### BBOX1-AS1 promotes HCC cell growth and migration

**Figure 2:**
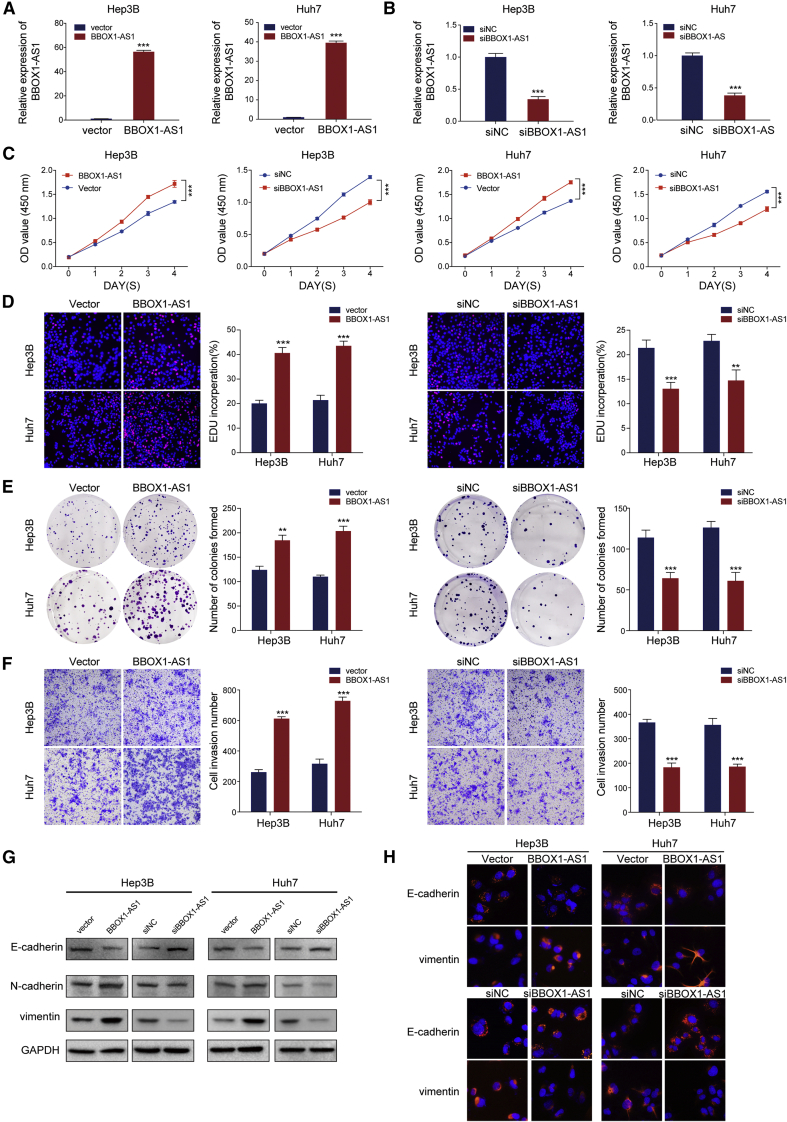
BBOX1-AS1 promotes proliferation and metastasis of HCC cells (A) The efficiency of BBOX1-AS1 overexpression in HCC cells was measured by qRT-PCR. (B) The efficiency of BBOX1-AS1 knockdown in HCC cells was measured by qRT-PCR. (C–E) CCK-8, EdU assays and colony formation assays were used to evaluate HCC cells proliferation after transfection. (F–H) Cell invasion and metastasis ability in transfected HCC cells were analyzed by Transwell, western blot, and immunofluorescence. ∗∗p < 0.01, ∗∗∗p < 0.001.

Nuclear-cytoplasmic RNA fractionation and FISH staining in cells showed that BBOX1-AS1 was predominantly located in the cytoplasm (Figures 1I and 1J). In order to verify that BBOX1-AS1 truly is non-coding RNA, ORF Finder from the National Center for Biotechnology Information was used to analyze the sequence, and the result showed that it failed to predict a protein of more than 80 amino acids (Figure S1A). Furthermore, both Coding-Potential Assessment Tool and Coding Potential Calculator analysis showed that the coding probability of BBOX1-AS1 was lower than 0.03, indicating that BBOX1-AS1 did not have protein-coding potential (Figures S1B and S1C). Based on RNA-seq data in ENCODE consortium, the expression of BBOX-AS1 variants in HepG2 was analyzed and visualized. (Figure S1D). To explore the biological functions of BBOX1-AS1 in HCC, BBOX1-AS1 was overexpressed and silenced in Hep3B and Huh7 cells (Figures 2A and 2B). Subsequent CCK-8 assays showed that the overexpression of BBOX1-AS1 remarkably strengthened the proliferative capacity of the transfected cells, whereas the silencing of BBOX1-AS1 had the opposite effect (Figure 2C). The effect of BBOX1-AS1 on cell proliferation in HCC was further verified by EdU and colony formation assays (Figures 2D and 2E). Furthermore, overexpression of BBOX1-AS1 enhanced cell metastasis, while the silencing of BBOX1-AS1 significantly inhibited the *in vitro* metastasis of HCC cells (Figure 2F). Then, western blot and immunofluorescence analysis further indicated that overexpression of BBOX1-AS1 promoted the EMT process, while BBOX1-AS1 silencing had the opposite effect in HCC cells (Figures 2G and 2H).

Taken together, the results revealed that BBOX1-AS1 facilitates the proliferation and metastasis of HCC.

### BBOX1-AS1 regulates sorafenib sensitivity of HCC through autophagy-related signaling

To explore the biological function of BBOX1-AS1 comprehensively, GO and KEGG enrichment analysis were conducted based on RNA-seq data. The results showed that BBOX1-AS1 might be involved in the regulation of autophagy and drug metabolism (Figures S2A and S2B).

**Figure 3:**
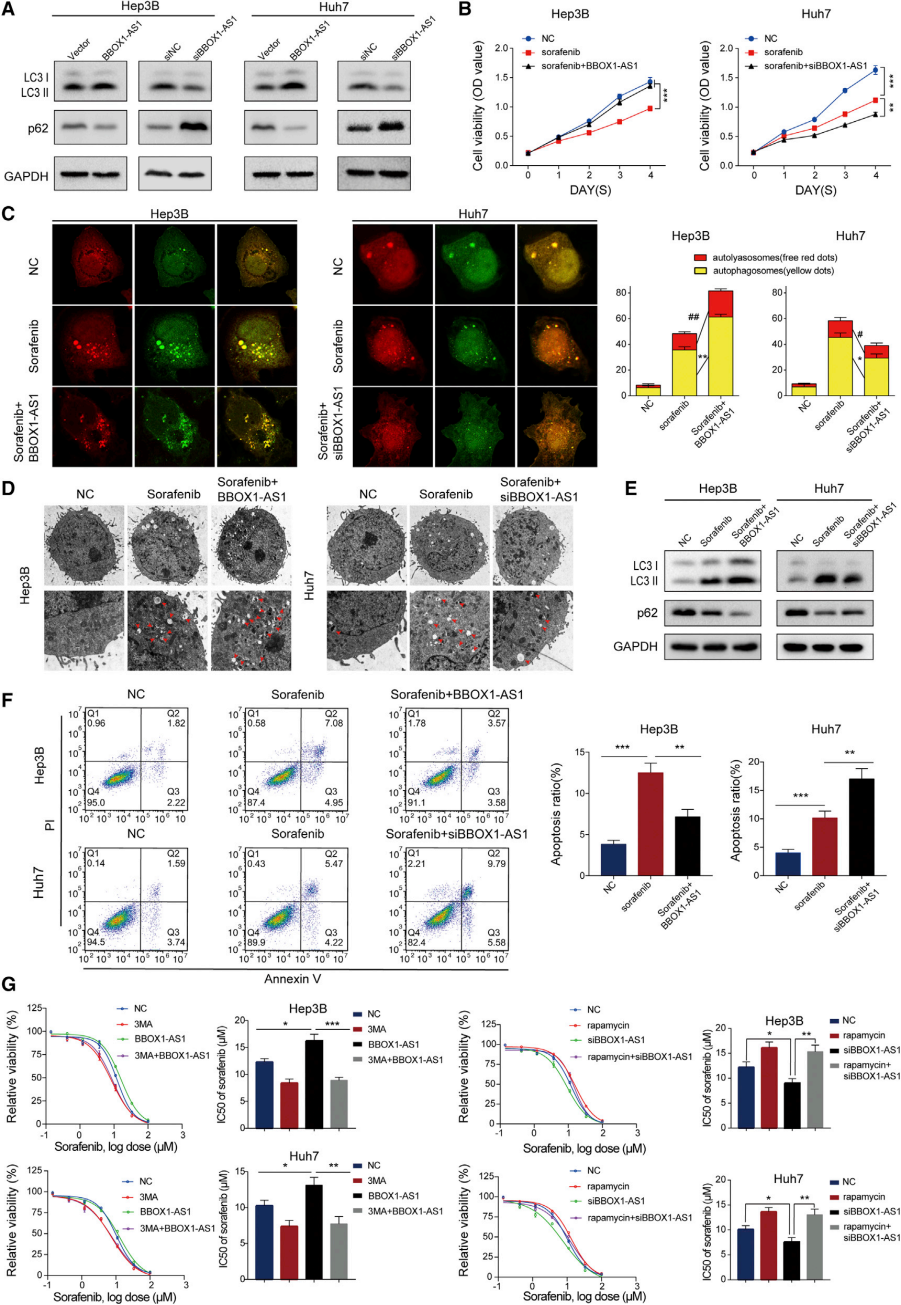
BBOX1-AS1 regulates autophagy and HCC cells sensitivity to sorafenib (A) Western blot was performed to detect the levels of autophagy-related proteins in HCC cells after transfection. (B) CCK-8 assays were performed to explore the role of BBOX1-AS1 in regulating sorafenib sensitivity in HCC (sorafenib: 10 μM). (C) Confocal microscopy was used to monitor the autophagosomes labeled by RFP-GFP-tagged LC3. Red dots indicate autolysosomes; yellow dots indicate autophagosomes (sorafenib: 10 μM). (D) TEM images were used to observe autophagosomes (sorafenib: 10 μM). (E) Western blot was performed to detect the role of BBOX1-AS1 and sorafenib in regulating autophagy (sorafenib: 10 μM). (F) Apoptosis assays were performed to explore the role of BBOX1-AS1 and sorafenib in cell apoptosis (sorafenib: 10 μM). (G) CCK-8 assays were performed to detect that 3-mA (2 mM) and rapamycin (10 nM) reverse the effect of sorafenib sensitivity induced by BBOX1-AS1 overexpression or knockdown. #p < 0.05, ##p < 0.01, ∗p < 0.05, ∗∗p < 0.01, ∗∗∗p < 0.001.

To explore the role of BBOX1-AS1 in autophagy, we analyzed the expression levels of autophagy-related proteins and found that BBOX1-AS1 expression was positively correlated with autophagy-related proteins (Figure 3A). Furthermore, BBOX1-AS1 could regulate sorafenib sensitivity by activating autophagy in HCC. CCK-8 assays showed that overexpression of BBOX1-AS1 remarkably weakened the cytotoxicity of sorafenib in HCC cells, while BBOX1-AS1 silencing enhanced the anticarcinogenic effect of sorafenib (Figure 3B). Subsequently, confocal microscopy images indicated that the numbers of autophagosomes increased markedly in HCC cells treated with sorafenib, and overexpression of BBOX1-AS1 further enhanced this effect. Conversely, the cells co-treated with sorafenib and siBBOX1-AS1 revealed significantly decreased numbers of autophagosomes compared with the cells treated with sorafenib alone (Figure 3C). Consistent with the confocal microscopy results, transmission electron microscopy (TEM) images and western blot assays revealed that the combination of BBOX1-AS1 overexpression and sorafenib promoted autophagy in HCC cells, whereas BBOX1-AS1 silencing weakened the effect of sorafenib on autophagy (Figures 3D and 3E). In addition, apoptosis analysis showed that BBOX1-AS1 overexpression reduced the sorafenib-induced apoptosis of HCC cells, while BBOX1-AS1 silencing had the opposite effect (Figure 3F). The autophagy inhibitor 3-MA (3-methyladenine, 3-MA) and the autophagy inducer rapamycin were used in sorafenib sensitivity assays. The results showed that 3-MA significantly attenuated the sorafenib resistance caused by BBOX1-AS1 overexpression, while rapamycin apparently reversed the sorafenib sensitivity induced by BBOX1-AS1 silencing (Figure 3G).

Therefore, we concluded that BBOX1-AS1 affects sorafenib sensitivity in HCC cells by regulating autophagy.

### BBOX1-AS1 upregulates PHF8 expression by sponging miR-361-3p in HCC cells

**Figure 4:**
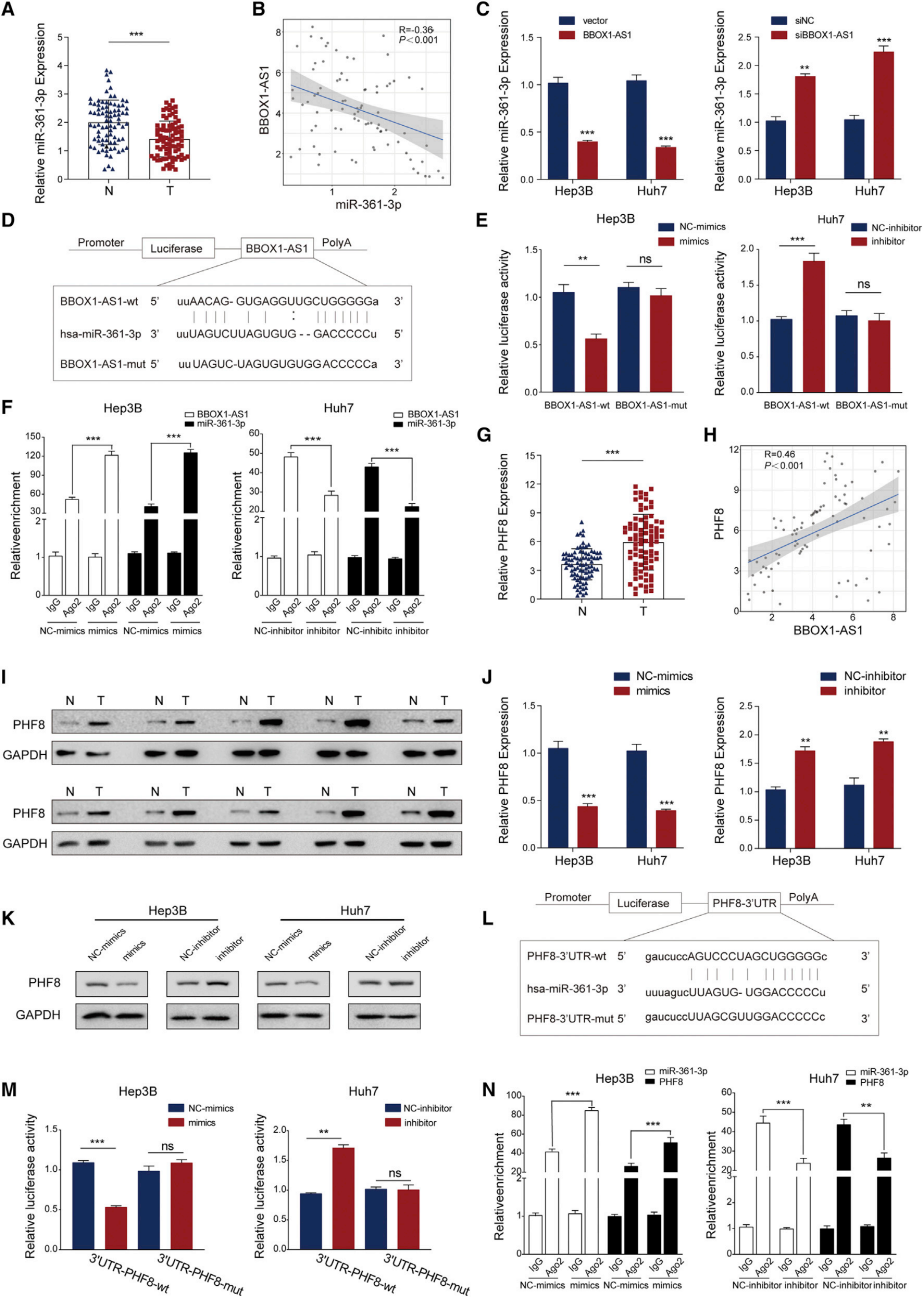
BBOX1-AS1 regulates autophagy and promotes HCC progression via regulation of PHF8 (A) qRT-PCR analysis of miR-361-3p expression in HCC tissues and matched adjacent normal tissues (n = 83). (B) Correlation analysis showing a negative correlation between miR-361-3p and BBOX1-AS1 expression. (C) qRT-PCR analysis of miR-361-3p expression in BBOX1-AS1 overexpression and silencing of HCC cells. (D) Schematic diagram of the binding sites between BBOX1-AS1 and miR-361-3p. (E) Dual-luciferase reporter assays analyzing the binding between BBOX1-AS1 and miR-361-3p. (F) RIP assays verified the binding between BBOX1-AS1 and miR-361-3p. (G) qRT-PCR analysis of PHF8 expression in HCC tissues and matched adjacent normal tissues (n = 83). (H) Correlation analysis showing a positive correlation between BBOX1-AS1 and PHF8 expression. (I) Western blot analysis of PHF8 expression in HCC tissues and matched adjacent normal tissues. (J and K) correlation between the PHF8 expression and miR-361-3p was analyzed in HCC cells by qPCR and WB. (L) Schematic diagram of the binding sites between miR-361-3p and PHF8-3′UTR. (M) Dual-luciferase reporter assays analyzing the binding between miR-361-3p and PHF8-3′UTR. (N) RIP assays verified the binding between miR-361-3p and PHF8 mRNA. ∗∗p < 0.01, ∗∗∗p < 0.001; ns, no significance. N, normal tissue; T, tumor tissue.

It has been reported that BBOX1-AS1 can regulate mRNAs by functioning as a ceRNA that sponges miRNAs in various cancers.^14^^,^^15^^,^^16^ According to the StarBase database, miR-361-3p was identified as a targeted miRNA of BBOX1-AS1. Then, we detected that miR-361-3p expression was markedly downregulated in HCC tissues (Figure 4A). Moreover, correlation analysis revealed a significant negative correlation between BBOX1-AS1 expression and miR-361-3p expression (Figure 4B). It was shown that BBOX1-AS1 overexpression decreased the expression level of miR-361-3p, while BBOX1-AS1 silencing had the opposite effect (Figure 4C). The transfection efficiency of miR-361-3p mimics and inhibitor were evaluated by qRT-PCR (Figure S2C). Luciferase reporter plasmids carrying BBOX1-AS1-wt and BBOX1-AS1-mut sequences were constructed according to the predicted binding site in StarBase (Figure 4D). The corresponding dual-luciferase reporter assays revealed that miR-361-3p mimics significantly decreased the relative luciferase activity, while miR-361-3p inhibitors increased the relative luciferase activity in BBOX1-AS1-wt groups, but the BBOX1-AS1-mut sequence abrogated these effects (Figure 4E). Moreover, RIP assays confirmed that Ago2 bound to BBOX1-AS1 and miR-361-3p, and the enrichment increased in the miR-361-3p mimic groups, but it decreased in the miR-361-3p inhibitor groups (Figure 4F).

Target miRNAs of BBOX1-AS1 were obtained through searching StarBase. PHF8 was screened as the potential target miR-361-3p through the intersection of downregulated mRNAs and predicted targets of miR-361-3p in StarBase (Figures S2D and S2E). Meanwhile, PHF8 was dramatically upregulated in HCC tissues, and its upregulation was associated with a poor prognosis (Figures 4G and S2F). Intriguingly, correlation analyses showed that the expression of PHF8 was negatively correlated with miR-361-3p expression but positively correlated with BBOX1-AS1 expression (Figures 4H and S2G). In addition, western blot analysis also further confirmed that PHF8 was upregulated in HCC tissues (Figure 4I). MiR-361-3p mimics decreased PHF8 expression, while miR-361-3p inhibitors increased the level of PHF8 in HCC cells (Figures 4J and 4K). Subsequently, PHF8-3′UTR-wt and PHF8-3′UTR-mut sequences were designed based on the predicted binding sequence in StarBase (Figure 4L). Dual-luciferase reporter assays showed that miR-361-3p mimics significantly decreased the relative luciferase activity, while miR-361-3p inhibitors enhanced the relative luciferase activity in the PHF8-3′UTR-wt groups. By contrast, these effects were not observed in the PHF8-3′UTR-mut groups (Figure 4M). In addition, RIP assays confirmed that PHF8 and miR-361-3p were present in RNA-induced silencing complexes (RISCs). The enrichment of RISCs was increased in the miR-361-3p mimic groups but decreased in the miR-361-3p inhibitor groups (Figure 4N).

In conclusion, the results indicated that BBOX1-AS1 functions as a ceRNA to sponge miR-361-3p, and PHF8 is a direct target of miR-361-3p.

### PHF8 is involved in BBOX1-AS1-modulated HCC progression and sorafenib resistance

**Figure 5:**
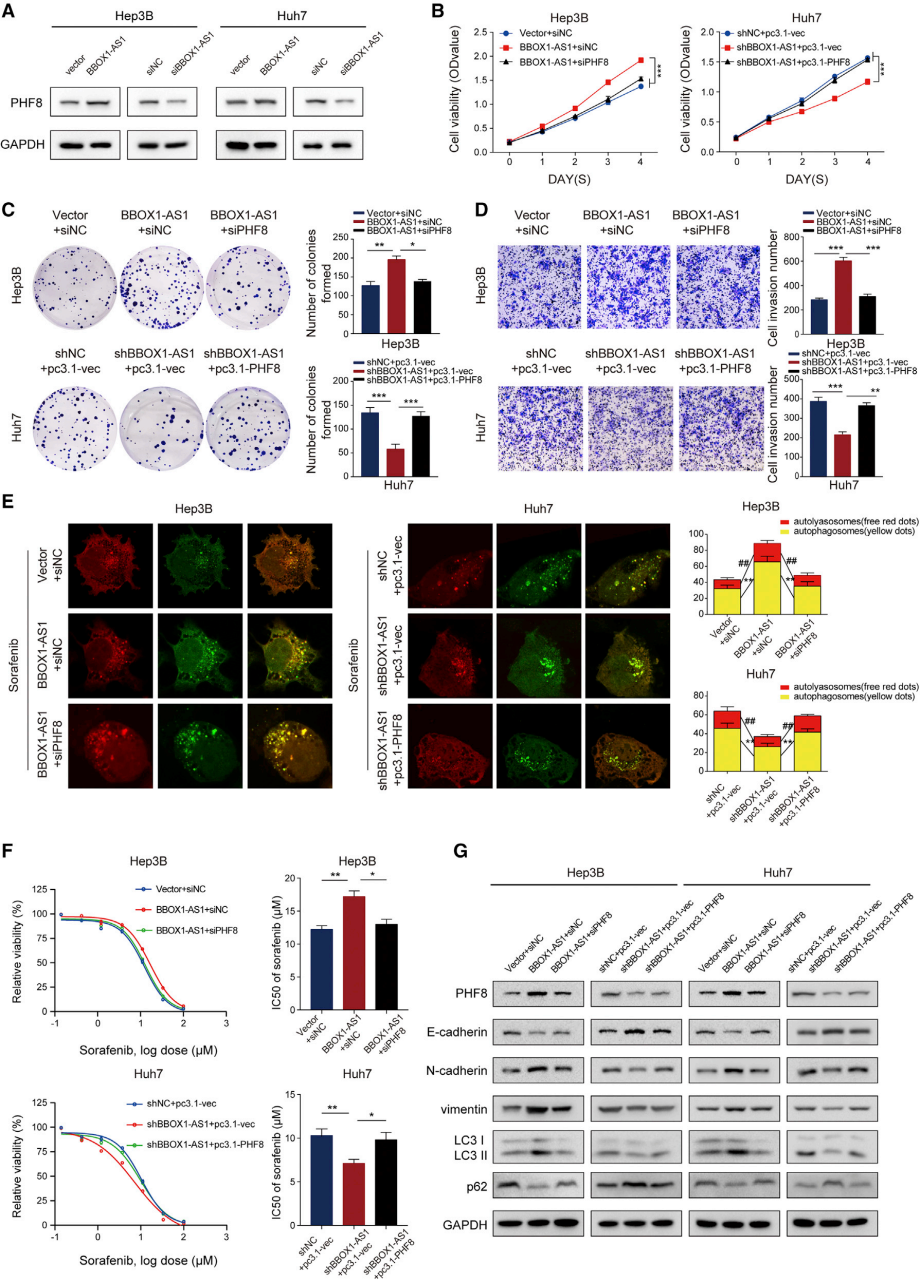
PHF8 is involved in BBOX1-AS1-modulated HCC progression and sorafenib resistance (A) Western blot was performed to detect that BBOX1-AS1 can regulate PHF8 expression level in HCC cells. (B and C) CCK8 and colony formation assays were performed to explore the ability of proliferation in HCC cells with indicated transfection. (D) Cell metastasis ability in transfected HCC cells was analyzed by Transwell assays. (E) Confocal microscopy was used to monitor the autophagosomes labeled by RFP-GFP-tagged LC3. Red dots indicate autolysosomes; yellow dots indicate autophagosomes (sorafenib: 10 μM). (F) Drug sensitivity assays were performed in HCC cells with indicated transfection. (G) EMT and autophagy-related proteins were detected by western blot. ##p < 0.01, ∗p < 0.05, ∗∗p < 0.01, ∗∗∗p < 0.001.

Further experiments were needed to determine if PHF8 is involved in BBOX1-AS1-modulated oncogenic activity. Western blot analysis confirmed that BBOX1-AS1 can regulate PHF8 expression in HCC cells (Figure 5A). To implement rescue assays, BBOX1-AS1-overexpressing cells were transfected with siPHF8, and BBOX1-AS1 downregulated cells were transfected with a PHF8-overexpression vector. The transfection efficiency was evaluated by qRT-PCR and western blot (Figures S3A and S3B). CCK8 and colony formation assays indicated that overexpression of BBOX1-AS1 facilitated cell proliferation, but the effect could be abrogated by PHF8 downregulation in HCC cells. At the same time, downregulation of BBOX1-AS1 inhibited the growth of HCC cells, while PHF8 overexpression restored proliferation (Figures 5B and 5C). Moreover, the effect on cell metastasis induced by BBOX1-AS1 overexpression could be counteracted by PHF8 silencing, but the cell metastasis inhibition caused by BBOX1-AS1 downregulation could be rescued by PHF8 overexpression (Figure 5D). Confocal microscopy images showed that the increase in the number of autophagosomes triggered by BBOX1-AS1 overexpression could be counteracted by PHF8 silencing, whereas overexpression of PHF8 restored the shBBOX1-AS1-induced decrease of autophagosome numbers (Figure 5E). Further drug sensitivity assays showed that PHF8 silencing significantly attenuated sorafenib resistance caused by BBOX1-AS1 overexpression, while upregulation of PHF8 could offset sorafenib sensitivity induced by BBOX1-AS1 silencing (Figure 5F). Likewise, western blot analysis also demonstrated that BBOX1-AS1 could modulate EMT and autophagy by regulating PHF8 (Figure 5G).

Taken together, the results indicate that BBOX1-AS1 modulates HCC progression and sorafenib resistance via PHF8.

### BBOX1-AS1 promotes HCC progression and sorafenib resistance through the miR-361-3p/PHF8 axis

**Figure 6:**
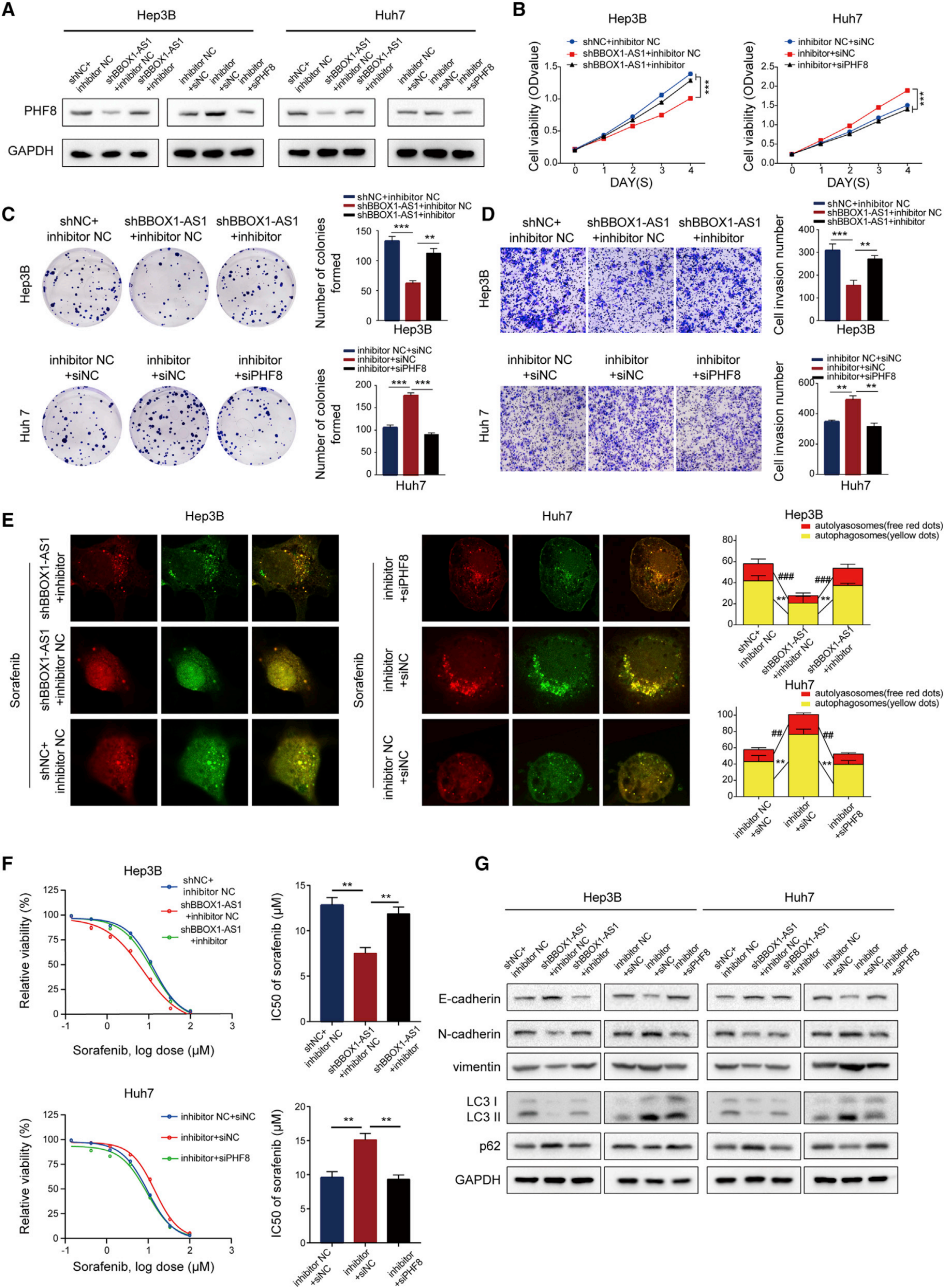
BBOX1-AS1 promotes HCC progression and sorafenib resistance through the miR-361-3p/PHF8 axis (A) Western blot was performed to detect that BBOX1-AS1 can regulate PHF8 expression level through the miR-361-3p/PHF8 axis. (B–D) CCK8, colony formation, and Transwell assays were performed to demonstrate that BBOX1-AS1 can promote cell proliferation and metastasis ability of HCC cells through the miR-361-3p/PHF8 axis. (E and F) Confocal microscopy images and drug sensitivity assays showed that BBOX1-AS1 can promote autophagy and sorafenib resistance of HCC cells through the miR-361-3p/PHF8 axis. (G) Western blot was performed to demonstrate that BBOX1-AS1 could modulate EMT and autophagy process through regulating miR-361-3p/PHF8. ##p < 0.01, ###p < 0.001, ∗∗p < 0.01, ∗∗∗p < 0.001.

To further validate whether BBOX1-AS1 promotes HCC progression and sorafenib resistance through the miR-361-3p/PHF8 axis, miR-361-3p inhibitor and siPHF8 were used to implement rescue experiments. Western blot analysis revealed that the decrease of PHF8 expression caused by BBOX1-AS1 knockdown could be rescued by the miR-361-3p inhibitor (Figure 6A). CCK8, colony formation, and Transwell assays indicated that the miR-361-3p inhibitor counteracted the suppression of cell proliferation and metastasis by BBOX1-AS1 knockdown. Conversely, the miR-361-3p inhibitor could promote cell proliferation and metastasis, which was reversed by PHF8 silencing (Figures 6B–6E). Moreover, confocal microscopy images and drug sensitivity assays revealed that the miR-361-3p inhibitor could attenuate the repression of autophagy and sorafenib resistance by BBOX1-AS1 knockdown. Conversely, PHF8 silencing inhibited the increase of autophagy and sorafenib resistance induced by the miR-361-3p inhibitor (Figure 6F). Similarly, western blot analysis also indicated that BBOX1-AS1 could modulate EMT and autophagy by regulating miR-361-3p/PHF8 (Figure 6G).

BBOX1-AS1 therefore promotes HCC progression and sorafenib resistance via the miR-361-3p/PHF8 axis.

### BBOX1-AS1 promotes HCC progression, and its downregulation increases sorafenib sensitivity *in vivo* and in organoid models

**Figure 7:**
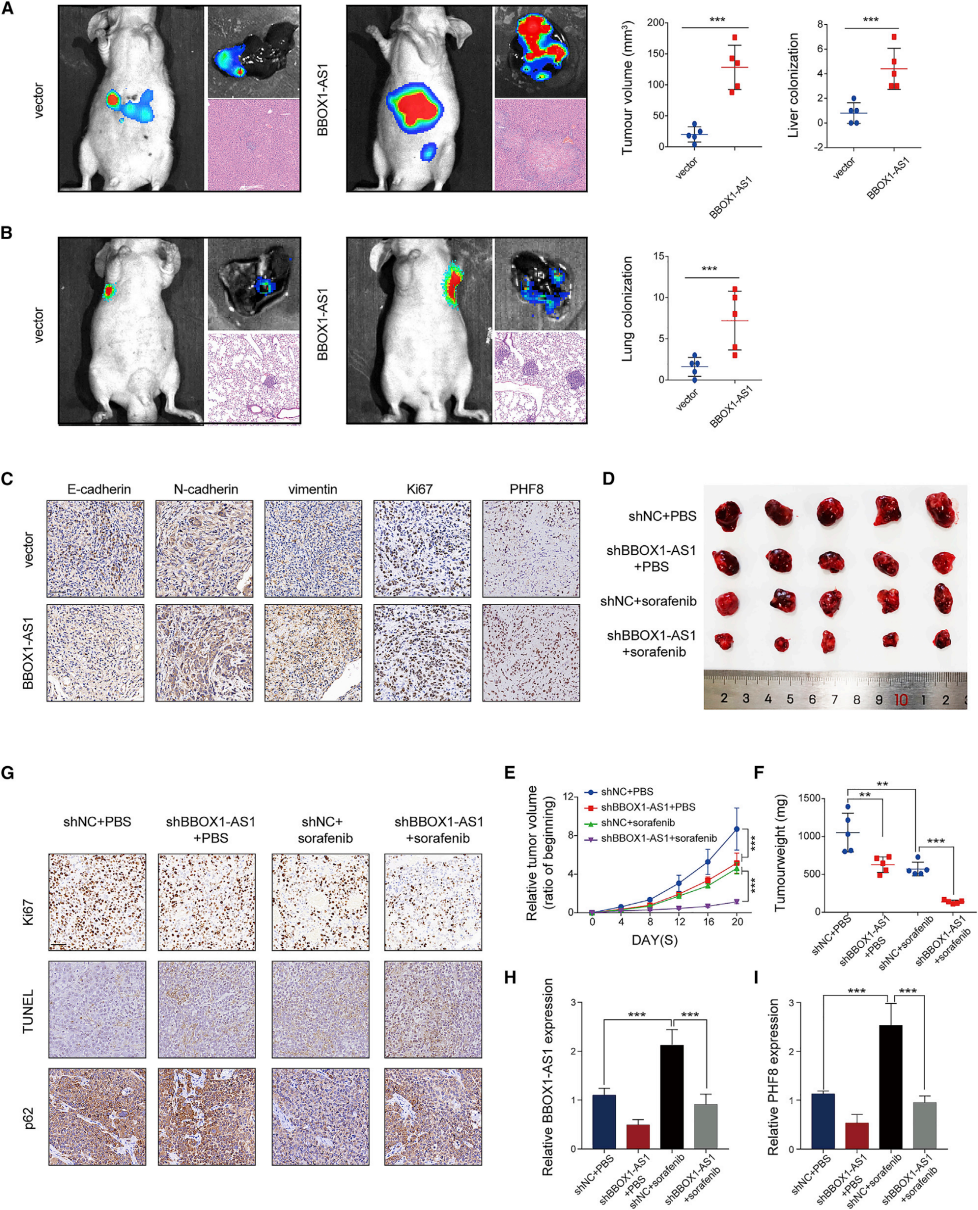
BBOX1-AS1 promotes HCC progression, and its downregulation increases sorafenib sensitivity *in vivo* (A) In orthotopic model mice, representative images (bioluminescent signal, gross and HE), as well as the size of the largest liver nodules and the number of liver metastatic nodules. (B) In lung metastasis model mice, representative images (bioluminescent signal, gross and HE), as well as the number of lung metastatic nodules. (C) Immunohistochemistry was applied to detect the expression of Ki67, PHF8, E-cadherin, vimentin, and N-cadherin in transfected cells. Scale bar: 50 μm. (D) Images of dissected tumors dissected after treatment with or without sorafenib in the presence or absence of BBOX1-AS1 knockdown. (E) Average percent change in tumor volume relative to the beginning for each treatment. (F) Weight of dissected tumors in each treatment group. (G) Representative pictures of immunohistochemistry for Ki67, TUNEL, and p62. Scale bar: 50 μm. (H and I) The relative expression levels of BBOX1-AS1 and PHF8 were analyzed in the four groups using qRT-PCR. ∗∗p < 0.01, ∗∗∗p < 0.001.

The effects of BBOX1-AS1 on HCC growth and metastasis were explored in orthotopic and lung metastasis models. In the orthotopic model, the BBOX1-AS1 overexpression group had larger tumors and more metastatic nodules in the liver (Figure 7A). In the lung metastasis model, the BBOX1-AS1 overexpression group had more and larger lung metastatic nodules (Figure 7B). In addition, immunohistochemistry results confirmed that upregulation of BBOX1-AS1 enhanced the expression of Ki-67, PHF8, N-cadherin, and vimentin, while it decreased E-cadherin expression (Figure 7C). Subsequently, subcutaneous HCC xenograft mouse models were established to further investigate the effect of BBOX1-AS1 on sorafenib resistance. The tumor volume and weight were remarkably decreased in the shBBOX1-AS1+PBS group and shNC + sorafenib group compared with the shNC + PBS group. Moreover, sorafenib treatment had a significantly greater inhibitory effect on the tumor growth of shBBOX1-AS1 transfected cells than on shNC cells (Figures 7D–7F). These results demonstrated that BBOX1-AS1 knockdown could enhance the efficacy of sorafenib *in vivo*. Immunohistochemical staining for p62 and Ki-67 further confirmed that BBOX1-AS1 knockdown led to significantly greater reduction of cell proliferation and cytoprotective autophagy in response to sorafenib treatment (Figure 7G). The relative expression of BBOX1-AS1 and PHF8 in four groups’ tumor samples demonstrated that BBOX1-AS1 knockdown effectively interfered with the promotion of BBOX1-AS1 and PHF8 induced by sorafenib (Figures 7H and 7I).

**Figure 8:**
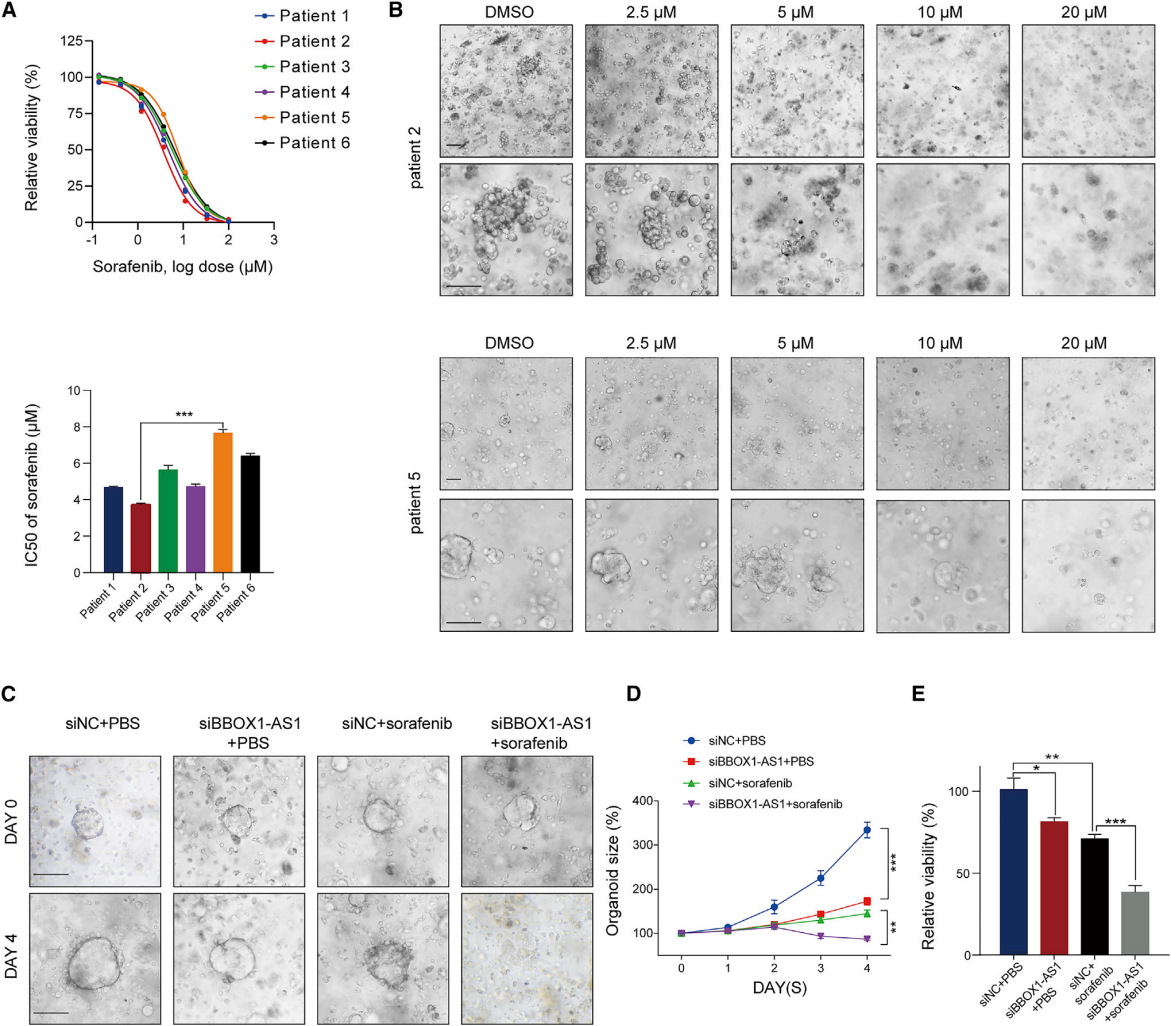
Downregulation of BBOX1-AS1 increases sorafenib sensitivity in patient-derived organoid models (A) Cell viability and IC50 were assessed for all six HCC patient organoids. (B) Representative light field images of sorafenib-treated HCC organoids (patients 2 and 5). Scale bar: 500 μm. (C) Light field images of organoids from patient 5 (days 0 and 4) with indicated treatment. (sorafenib: 8 μM). Scale bar: 500 μm. (D) Organoid size change of organoids with indicated treatment (relative to the beginning for each treatment). (E) Cell viability of organoids with indicated treatment on day 4. ∗p < 0.05, ∗∗p < 0.01, ∗∗∗p < 0.001.

Furthermore, HCC organoid models were built to investigate the effect of BBOX1-AS1 on sorafenib resistance. The results showed that organoids from different patients had different IC_50_ values, which varied from 3.72 to 7.65 μM (Figure 8A). Among them, organoids from patient 5 with the highest IC_50_ value were obviously more resistant to sorafenib than organoids from patient 2 (Figure 8B). Subsequent qPCR analysis showed that BBOX1-AS1 was significantly upregulated in organoids from patient 5 compared with those from patient 2 (Figure S3C), indicating that tumors with high BBOX1-AS1 expression might be prone to sorafenib resistance. Then, we further verified the effect of BBOX1-AS1 on HCC progression and sorafenib resistance in organoids from patient 3 (Figure 8C). These data indicated that both sorafenib and BBOX1-AS1 knockdown could inhibit the growth of HCC organoids, while a combination of BBOX1-AS1 knockdown with sorafenib had a stronger killing effect than sorafenib alone (Figures 8D and 8E).

In summary, we demonstrated the role of BBOX1-AS1 in promoting HCC progression and sorafenib sensitivity in xenografted HCC tumors in mice *in vivo* and in organoid models.

## Discussion

HCC is a common malignant tumor with complex cancer biology and high worldwide incidence.^17^ Most patients with HCC are diagnosed at an advanced stage and have a poor prognosis.^18^ One significant factor for the adverse prognosis of advanced-stage HCC patients is acquired drug resistance during treatment, and its underlying mechanism has not been clearly elucidated.^19^ Recent studies indicate that some oncogenic ncRNAs are associated with acquired drug resistance.^2^ For example, lncRNA CRNDE can participate in the regulation of multiple molecular regulatory networks in a number of cancer types, including kidney,^20^ pancreatic,^21^ colorectal,^22^ and liver cancer.^23^ CRNDE is also a key factor in radio and chemoresistance, including sorafenib resistance in HCC.^24^^,^^25^ Additionally, recent studies suggest that some lncRNAs exhibit a remarkable HCC-specific expression.^26^^,^^27^^,^^28^ The candidate lncRNAs have been proven to be involved in HCC progression or metastasis and may be suitable as a prognosis and metastasis biomarker in HCC patients.^28^ Therefore, approaches targeting ncRNAs that are both associated with HCC progression and sorafenib resistance may facilitate the development of promising strategies for treating HCC.

Numerous studies have demonstrated that BBOX1-AS1 is highly expressed in various cancers, including HCC.^14^^,^^15^^,^^16^ However, there are still no studies analyzing the biological function and mechanism of BBOX1-AS1 in HCC progression. In this study, BBOX1-AS1 expression was found to be significantly positively correlated with PHF8, which could promote the EMT process and autophagy in HCC. Therefore, we investigated whether BBOX1-AS1 is involved in the regulation of PHF8 and autophagy in HCC. First, BBOX1-AS1 was confirmed to promote the growth and migration in HCC cells. Then, BBOX1-AS1 participates in the regulation of autophagy in HCC cells. Autophagy is an evolutionarily conserved intracellular degradation process that is involved in the maintenance of cellular homeostasis and cellular stress responses.^25^^,^^29^ Numerous studies have highlighted that autophagy plays key roles in multiple biological processes crucial for HCC development, including proliferation, metastasis, and sorafenib resistance.^30^ Thus, we further explored whether BBOX1-AS1 could regulate sorafenib sensitivity in HCC. The present study indicated that BBOX1-AS1 promoted sorafenib resistance in HCC cells by activating cytoprotective autophagy.

PHF8, also known as KDM7B, is a histone demethylase that acts as a transcription coactivator. PHF8 is highly expressed and associated with cancer progression and metastasis of diverse cancers.^31^^,^^32^ Additionally, PHF8 was identified as an oncogenic protein in HCC and a key molecular regulator of EMT and autophagy.^33^ In the Tongji cohort, PHF8 expression was correlated with BBOX1-AS1 expression and HCC prognosis. Then, a series of rescue assays revealed that PHF8 knockdown could partially weaken the biological effects of BBOX1-AS1 overexpression, while PHF8 upregulation could rescue the effects of BBOX1-AS1 silencing. These results demonstrated that BBOX1-AS1 promoted HCC progression and cytoprotective autophagy by upregulating PHF8. Subsequently, it was necessary to further explore the underlying mechanism through which BBOX1-AS1 regulates PHF8 in HCC. There is accumulating evidence that RNA stability could be affected by multiple factors such as ribonucleases, RNA binding proteins, and miRNAs.^34^ BBOX1-AS1 is mostly located in the cytoplasm, indicating that cytoplasmic BBOX1-AS1 may function as a ceRNA that affects targeted miRNAs. Recent studies have shown that BBOX1-AS1 acts a sponge that regulates a number of miRNAs, including miR-3940-3p, miR-27a-5p, and miR-361-3p.^14^^,^^15^^,^^16^ It has been reported that miR-361-3p exhibited low expression and acted as a tumor suppressor gene in multiple cancers, including HCC.^35^^,^^36^ Our study demonstrated that miR-361-3p might directly bind to BBOX1-AS1. In the Tongji cohort, miR-361-3p was significantly downregulated in HCC tissues and negatively correlated with BBOX1-AS1. Subsequent experiments showed that PHF8 might be the targeted mRNA of miR-361-3p. Taken together, the data indicate that BBOX1-AS1 acts as a sponge for miR-361-3p in HCC cells. Finally, a series of rescue experiments were implemented, and the results confirmed that BBOX1-AS1 promotes HCC progression and sorafenib resistance via the miR-361-3p/PHF8 axis. *In vivo* xenograft experiments showed that BBOX1-AS1 overexpression could promote the proliferation and metastasis of HCC. Moreover, sorafenib treatment *in vivo* assays showed that BBOX1-AS1 knockdown could enhance the efficacy of sorafenib in HCC. Due to high heterogeneity of HCC, cell lines and animal experiments cannot fully reflect the heterogeneous tumor background of patients and predict the clinical outcomes of different individuals.^37^ Thus, the results obtained in cell lines and animal experiments have substantial limitations. Patient-derived organoids that preserve tumor heterogeneity to some extent can overcome these limitations, which makes them invaluable preclinical models for cancer research. Similar experiments conducted on patient-derived organoids further confirmed that BBOX1-AS1 knockdown not only inhibited tumor progression but also effectively restrained sorafenib resistance in HCC. Therefore, approaches designed to target BBOX1-AS1, which is associated with both tumor progression and sorafenib resistance, may provide a highly promising treatment strategy for HCC.

One weak point of the present study is that we only detected BBOX1-AS1 in HCC tissue samples obtained through surgical resection, but we did not detect its levels in the plasma, saliva, and other bodily fluids. In fact, circulating cell-free ncRNAs and circulating exosomes have been detected and confirmed to be closely associated with tumor development, metastasis, and drug sensitivity. In a following study, we will continue to explore whether the abovementioned ncRNAs can be found in the serum, plasma, or exosomes of patients and thus may be used for liquid biopsy.

### Conclusions

In summary, we identified BBOX1-AS1 as a novel oncogenetic lncRNA that promotes the proliferation and metastasis of HCC. Furthermore, we for the first time revealed that BBOX1-AS1 is also involved in autophagy regulation and could modulate sorafenib sensitivity by activating cytoprotective autophagy in HCC. Mechanistically, BBOX1-AS1 promoted tumor progression and sorafenib resistance by regulating miR-361-3p/PHF8. The results of experiments performed on xenografted tumors in nude mice and patient-derived organoids confirmed that BBOX1-AS1 knockdown could effectively block cancer progression and sorafenib resistance in HCC. Thus, approaches designed to target BBOX1-AS1 may provide a powerful novel therapeutic strategy for the treatment of HCC.

## Materials and methods

### Patients and tissue specimens

A total of 83 pairs of HCC tissues and adjacent non-tumor tissues were obtained from HCC patients who underwent surgery between 2014 and 2016 at the Hepatic Surgery Center of Tongji Hospital (Wuhan, China). The detailed information of all cancer cases is summarized in Table S1. Inclusion criteria of HCC patients were as follows: definite HCC diagnosis based on pathology in accordance with WHO criteria, with frozen tissues, and with complete follow-up data. All experiments involving patient samples were approved by the Ethics Committee of Tongji Hospital (Approval No. TJ-IRB20210924).

### Autophagic flux assay

To monitor the autophagic flux, we used mRFP-GFP-LC3 adenovirus (DesignGene Biotechnology, China) to transfect HCC cells and track LC3. First, Huh7 and Hep3B cells (1 × 10^5^ cells/well) were cultured on cover slips in 24-well plates for 12 h. Following 24 h after mRFP-GFP-LC3 adenovirus transfection, the cells were subjected to different treatments for an additional 48 h. Subsequently, the treated cells were fixed with 4% paraformaldehyde for 30 min and observed by confocal laser scanning microscopy (Leica, Germany). Yellow fluorescent spots (overlay of GFP signal and mRFP signal) represented early autophagosomes, while red fluorescent spots (mRFP signal alone) represented late autolysosomes. The changes in the average number of autolysosomes and autophagosomes were quantified to evaluate autophagic flux.

### Transmission electron microscopy

HCC cells were fixed in 0.1 M sodium dimethyl arsenate solution containing 2.5% glutaraldehyde at 4°C overnight. Then, the cells were post-fixed in 1% osmic acid at 4°C for 1.5 h, washed with PBS, dehydrated with a gradient of increasingly concentrated ethanol solutions, and embedded in acrylic resin at 60°C for 48 h. The 70-nm ultrathin sections were mounted on a nickel grid. The samples were stained with uranyl acetate for 20 min, followed by lead citrate for 10 min, and then rinsed with distilled water. The samples were observed and images recorded using a 120-kV HT7700 transmission electron microscope (Hitachi, Japan).

### Animal experiments

Male BALB/c nude mice (4 weeks old) were maintained under specific-pathogen-free conditions. To construct the orthotopic model, 2 × 10^6^ luciferase-bearing HCC cells in 30 μL serum-free DMEM were injected into the left hepatic lobe of nude mice. To evaluate the tumor metastasis *in vivo*, 2 × 10^6^ luciferase-expressing HCC cells were injected into the tail veins of mice. Tumor growth and metastasis were monitored using an IVIS Lumina XRMS Series III live imaging system (PerkinElmer, USA), and all mice were sacrificed 8 weeks after xenografting. For the sorafenib treatment assay, 2 × 10^6^ cells in 100 μL serum-free DMEM were injected subcutaneously into the flanks of nude mice. After successful tumor formation, the mice were orally administered the indicated treatments by gavage every day during the 14 days. Sorafenib was administered at a dosage of 50 mg/kg, suspended in DMSO (Sigma, USA) and corn oil (MCE, USA) at a ratio of 1:9. The volumes were measured every 4 days, and tumor weights were measured after the mice were sacrificed. The tumor volume was calculated according to the formula, volume (mm^3^) = 0.5 × L (length, mm) × W^2^ (width, mm^2^). The whole procedure was performed following the "Guide for the Care and Use of Laboratory Animals" (NIH publication 86–23, revised 1985) and was approved by the Committee on the Ethics of Animal Experiments of Tongji Hospital.

### Construction of HCC organoid models

HCC tissues were rinsed with cold PBS and macerated on ice. Tissue fragments were then digested with collagenase IV (2 mg/mL, Sigma) and DNase I (0.1 mg/mL, Sigma) at 37°C for 15 min on an orbital shaker. Then, advanced DMEM (Gibco, USA) with 10% FBS was added to stop the digestion, after which the suspension was collected and pelleted by centrifugation at 300 *g* for 5 min. After repeated washing with cold PBS by centrifugation, the cells were counted, mixed with Matrigel (Corning, USA), and seeded into 24-well plates. After the Matrigel solidified within 30 min, fresh organoid culture medium was added into the 24-well plates. The composition of the organoid culture medium is summarized in supplemental materials and methods. The growth of HCC organoids was observed daily under a microscope. According to the manufacturer’s instructions, CellTiter-Glo 3D reagent (Promega, USA) and a GloMax luminescence detector (Promega) were used to examine organoids viability. HCC organoids were transfected with BBOX1-AS1 siRNA or siNC and then used to confirm the role of BBOX1-AS1 in promoting HCC progression and sorafenib sensitivity.

### Statistical analysis

We used SPSS 22.0 (IBM, USA) and Prism 7.0 (GraphPad, USA) to perform statistical analyses. Quantitative data were analyzed using two-tailed Student’s t test or one-way ANOVA. The Chi-squared (χ^2^) test was used for comparisons of categorical data. Pearson’s correlation coefficient was used to analyze linear correlations. Overall survival was evaluated using Kaplan-Meier survival curves. Statistical tests and p values were two sided. Differences with p < 0.05 were considered statistically significant.

Supplemental materials and methods are shown in supplemental information.

## Data Availability

The data that support the findings of this study are available on request from the corresponding author.

## Supplemental information

Document S1. Tables S1–S4, Figures S1–S3, and supplemental materials and methods

Document S2. Article plus supplemental information

## Footnotes

Supplemental information can be found online at https://doi.org/10.1016/j.omto.2022.12.005.
