# Supplementary material for: Oncogenic lncRNA BBOX1-AS1 promotes PHF8-mediated autophagy and elicits sorafenib resistance in hepatocellular carcinoma
Source: Mol Ther Oncolytics. 2022 Dec 23;28:88–103. doi: 10.1016/j.omto.2022.12.005 (PMC9852557; doi:10.1016/j.omto.2022.12.005)
Supplement: Document S1. Tables S1–S4, Figures S1–S3, and supplemental materials and methods [file mmc1.pdf]

OMTO, Volume *28*

Supplemental information

Oncogenic lncRNA BBOX1-AS1 promotes PHF8-mediated autophagy and elicits sorafenib resistance in hepatocellular carcinoma

Haisu Tao, Yuxin Zhang, Jiang Li, Junjie Liu, Tong Yuan, Wenqiang Wang, Huifang Liang, Erlei Zhang, and Zhiyong Huang

Supplemental Tables

Table S1. Associations between BBOX1-AS1 and clinicopathological parameters in HCC.

Variables	BBOX1-AS1 (low) n=41	BBOX1-AS1 (high) n=42	P value
Age,years, ≥50/ < 50	19/22	24/18	0.325
Sex,male/female	18/23	21/21	0.578
ALT,U/L, < 40/≥40	24/17	21/21	0.435
AST,U/L, < 40/≥40	18/23	25/17	0.154
AFP,ng/mL, < 55/≥55	19/22	15/27	0.325
HBsAg,Negative/Positive	11/30	11/31	0.947
Encapsulation,Absent/Present	18/23	24/18	0.228
Tumor differentiation, Well/Moderate-Poor	10/31	7/35	0.383
Vascular invasion, No/Yes	28/13	14/28	0.001**
Tumor size, cm, < 5/≥5	15/26	19/23	0.423
Tumor number, Single/Multiple	37/4	32/10	0.087
BCLC stage, A/B-C	26/15	23/19	0.423
TNM, Ⅰ-Ⅱ/Ⅲ-Ⅳ	28/13	16/26	0.006**
Liver cirrhosis,yes/no	21/20	25/17	0.447

**p < 0.01

Table S2. Interfering oligonucleotides sequence used in this study.

Name	Sequence	Company
siBBOX1-AS1	GAAGTTTCTTTCAAACTCTGC	RiboBio
shBBOX1-AS1	CCGGGAAGTTTCTTTCAAACTCTGCCTCGAG GCAGAGTTTGAAAGAAACTTCTTTTT	Vigene Biology
siPHF8	GCTCTTTCCAGAAAGCAAAGT	RiboBio
miR-361-3p mimics	UCCCCCAGGUGUGAUUCUGAUUU	RiboBio
miR-361-3p inhibitor	AAAUCAGAAUCACACCUGGGGGA	RiboBio

Table S3. Primers sequence used in this study.

Primers used for quantitative RT-PCR		
**Name**	**Forward-primer**	**Reverse-primer**
BBOX1-AS1	CCTGAATACCAAAGAGGGCCG	TGAAGCCTCTCTCTGCTAGGT
PHF8	GTGCCGGTGTATTGCCTCT	CAACACAACTGCCATGAAACC
miR-361-3p	CTCCCCCAGGTGTGATTCTGATTT	
GAPDH	CTGGGCTACACTGAGCACC	AAGTGGTCGTTGAGGGCAATG
U6	CTCGCTTCGGCAGCACA	AACGCTTCACGAATTTGCGT

Table S4. Antibodies used in this study.

Name	Company	Catalog Number
N-cadherin	Proteintech	#22018-1-AP
Vimentin	ABclonal	#A11952
E-cadherin	Proteintech	#20874-1-AP
LC3B	Cell Signaling Technology	#2775
P62	Cell Signaling Technology	#23214
PHF8	ABclonal	#A19780
Ki-67	Proteintech	#27309-1-AP
Ago2	Abcam	Ab186733
GAPDH	Proteintech	#60004-1-lg

Supplemental Figures

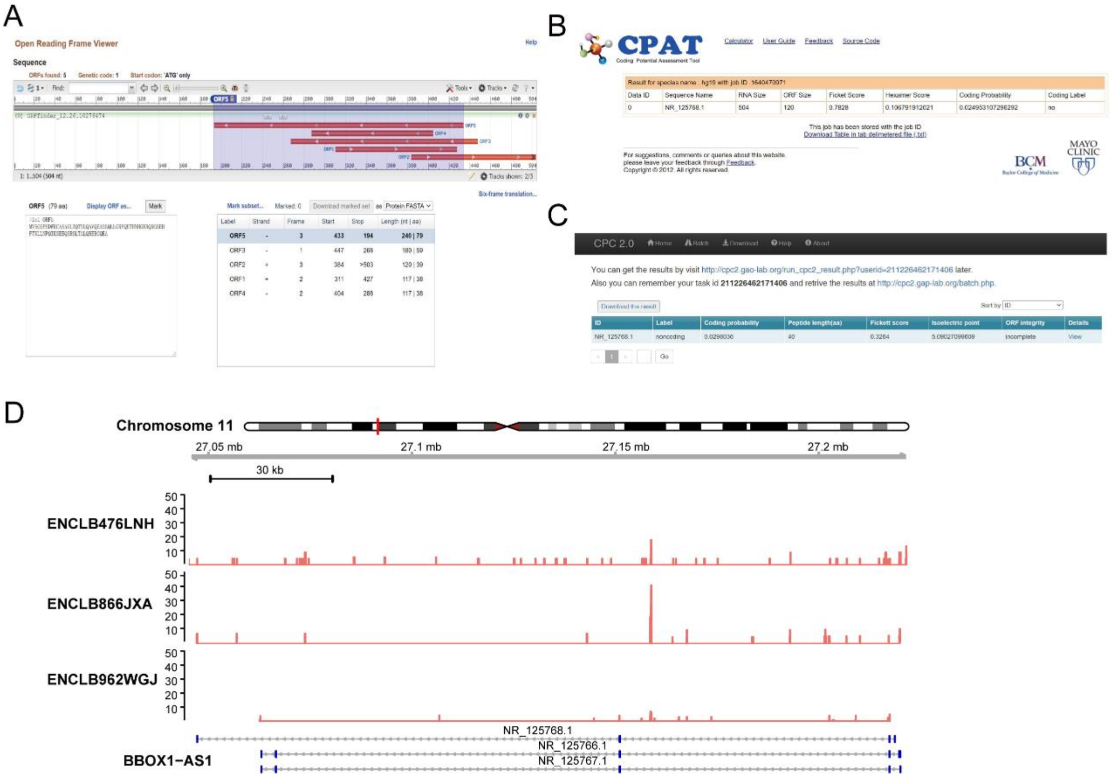

Fig S1.

A, The analysis results of BBOX1-AS1 in ORF Finder.

B, The analysis results of BBOX1-AS1 in CPAT.

C, The analysis results of BBOX1-AS1 in CPC.

D, Based on RNA-seq data in ENCODE consortium, the expression of BBOX-AS1 variants in HepG2 was analyzed and visualized.

Fig. S2

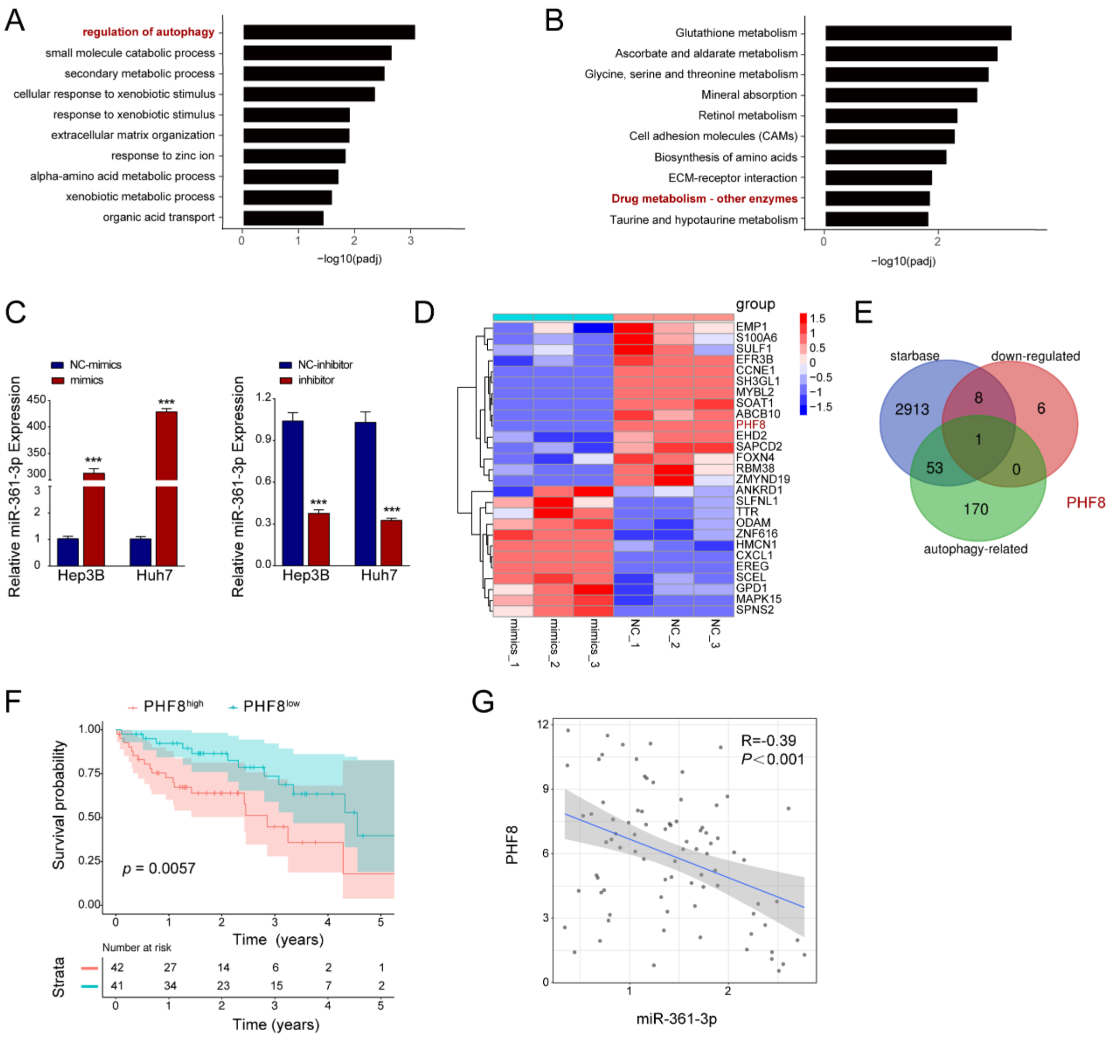

Fig S2.

A, GO biological processes analysis of the BBOX1-AS1 regulated genes. B, KEGG analysis of the BBOX1-AS1 regulated genes. C, The transfection efficiency of miR-361-3p mimics and inhibitor were evaluated by qRT-PCR. D, The heat map of differentially expressed mRNAs in miR-361-3p overexpressing cells. E, The Venn diagram showed the overlaps of the analysis. F, Kaplan-Meier survival curve was used to explore the effect of PHF8 on overall survival (n = 83). G, Correlation analysis showing a negative correlation between miR-361-3p and BBOX1-AS1 expression. ***P < 0.001

Fig. S3

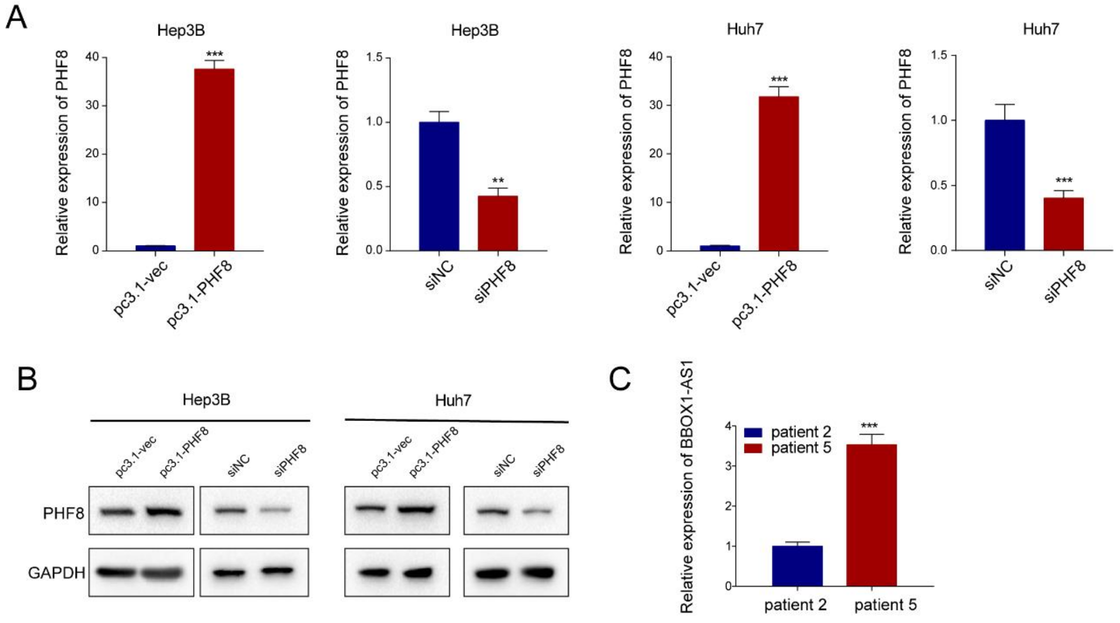

Fig S3.

A, The efficiency of silenced and overexpressed PHF8 in HCC cells was measured by qRT-PCR. B, The efficiency of silenced and overexpressed PHF8 in HCC cells was measured by western blot. C, The relative level of BBOX1-AS1 in organoids from patient 5 and patients 2 was evaluated by qRT-PCR. **P < 0.01, ***P < 0.001

Supplemental Materials and Methods

Cell culture

Normal-type hepatocyte HL7702, hepatoma cell line HepG2, human HCC cell lines 97H, HLF, LM3, Huh7 and Hep3B and the lentivirus packaging cell line HEK-293T were purchased from China Center for Type Culture Collection (CCTCC, China). All cell lines were cultured in high-glucose DMEM (HyClone,USA) supplemented with 10% FBS at 37°C in an atmosphere of 5% CO_2. The cells in the sorafenib treatment groups were maintained in an environment with the drug concentration of 10 μM or the indicated concentrations of sorafenib.

Cell transfection

The pcDNA3.1 vector containing the full-length cDNA sequences of PHF8 and short hairpin RNAs (shRNAs) against BBOX1-AS1 were provided by Tsingke Biological Technology (Beijing, China). The pcDNA3.1 empty vector and scramble shRNA or siRNA were utilized as negative controls. Lentivirus particles containing BBOX1-AS1 and PHF8 were ordered from DesignGene Biotechnology (Shanghai, China). SiBBOX1-AS1, siPHF8, miR-361-3p mimics and miR-361-3p inhibitors were designed by RiboBio (Guangzhou, China). To construct luciferase-bearing HCC cells, cell lines were transfected with PGL4.51 plasmid (Promega, USA) as described in our previous study[1]. According to the manufacturer's instructions, when cells reach 70% density, the cell transfections were carried out using Lipo 3000 Transfection Reagent (Invitrogen). The interfering oligonucleotides sequence are shown in Supplementary Table 2.

Quantitative real-time PCR assay

Total RNA was extracted from tissues or cell lines using TRIzol reagent (Invitrogen, USA) and cDNA of lncRNA and mRNA were synthesized from the total RNA using the HiScript®II Q RT SuperMix for qPCR (Vazyme, China). For miRNA, reverse transcriptions were performed using the Mir-X miRNA First-Strand Synthesis Kit (Takara, Japan). Real time PCR was performed using SYBR Green PCR kit (Vazyme, China). GAPDH and U6 were used as the endogenous control. The relative expression levels of the genes were calculated by comparing to GAPDH or U6 level using $2-\Delta\Delta CT$ method. The primers are shown in Supplementary Table 3.

Western blot

Proteins from tissues and cells were extracted with RIPA buffer containing a protease inhibitor cocktail (MedChemExpress, USA). Following protein extraction, protein concentrations were determined using a BCA Protein Assay Kit (Promega, USA). Equal amounts (20 μg/lane) of protein were analyzed by 10% SDS-PAGE (Yeasen, China) and transfer to PVDF membranes (Millipore, USA). Subsequently, the membranes were blocked with 5% non-fat milk at 37 °C for 1 h and were incubated with primary antibodies at 4 °C overnight. Next, these membranes were incubated with horseradish peroxidase-conjugated secondary antibodies (Jackson ImmunoResearch, USA) at 37 °C for 2 h. Finally, the protein bands were visualised with ECL detection

system (Bio-Rad Laboratories, USA). Information on the antibodies is shown in Supplementary Table 4.

EdU, Cell Counting Kit-8 assay and colony formation assay

For EdU assay, cells were stained using a Cell-Light EdU staining kit (RiboBio, China) according to its instructions. After that, a fluorescence microscope was used to take pictures, with 3 fields randomly selected for each slide. Lastly, the number of EdU-positive cells was counted and quantified. Cell proliferation ability was also measured using the Cell Counting Kit-8 (CCK-8, Beyotime Institute of Biotechnology, China). Cells were plated in 96-well plates at the indicated density and cultured in complete medium. The absorbance at 450 nm of each sample was measured 2 h later at 37 °C using a microplate reader at a test wavelength of 450 nm(Bio-Tek, USA). Regarding colony formation experiment, 1000-2000 cells were seeded in each well of 6-well plates, allowing cells to grow until visible colonies formed. The colonies were fixed and stained with a dye solution containing 0.1% crystal violet and 20% methanol, and the number of colonies was counted.

Transwell invasion assay

For the invasion assay, cells (5×10^4 Hep3B cells and 3×10^4 Huh7 cells for migration assays) were seeded in 24-well Transwell plates (Corning, USA), according to the manufacturer's protocol. The membranes of the inserts were pre-coated with Matrigel matrix at 24 h prior to cell seeding. Serumfree tumor cells in serum-free medium were placed in the upper chamber and culture medium containing 10% FBS was placed in the lower chamber. After a 24 h incubation at 37 °C, the non-invading or non-migration cells on the upper membrane were removed mechanically. Cells on the lower surface of the membranes were fixed with 4% paraformaldehyde for 15 min and stained with 0.1% crystal violet for 2 h. For visualization, the cells were photographed and counted from five random fields.

Apoptosis analysis

Cells were seeded into six-well plates at a density of 1×10^5 cells per well and incubated with 10 μmol/L sorafenib for 24h. Then, cells were stained with propidium iodide (PI) and Annexin V-FITC Apoptosis Detection Kit I (BD, USA) according to manufacturer’s protocol. Finally, the cells were measured by FACS (BD, USA) for apoptosis and Cell Quest Research Software (BD, USA) was used to analyze data.

Fluorescence in situ hybridization (FISH)

Cells were cultured on coverslips in 24-well plates. Cy3-labeled BBOX1-AS1 detection probe was purchased from Ribobio (Guangzhou, China). RiboTM FISH Kit (Ribobio, China) was used to perform hybridization steps in HCC cells and tissues. Images were taken and analyzed with confocal laser scanning microscopy (Leica, Germany).

RNA immunoprecipitation (RIP)

Magna RIP™ RNA-Binding Protein Immunoprecipitation Kit (Millipore, USA) was used to perform RIP assay. Briefly, cells with indicated transfection were harvested and lysed by complete RIP lysis buffer. Then, cell lysates were incubated with beads which were pre-coated with Ago-2 antibody or anti-IgG antibody overnight at 4°C. Finally, enriched RNA was extracted with TRIzol after being treated with DNase and Proteinase K and then analyzed by qRT-PCR.

Luciferase reporter assay

The sequences of BBOX1-AS1 (BBOX1-AS1-wt), mutated BBOX1-AS1 (BBOX1-AS1-mut), 3′-untranslated region (UTR) of the PHF8 (3'UTR-PHF8-wt) and mutated 3'UTR of the PHF8 (3'UTR-PHF8-mut) were cloned into the luciferase reporter vector psiCHECK-2 (Promega, USA). Firstly, Huh7 and Hep3B cells (2×10^5/ well) were seeded in 24-well plates for 12 h. Then, the cells were co-transfected with 50 ng of each psiCHECK-2 vector, and 50 nM of the miR-361-3p mimic, miR-361-3p inhibitor or their negative control (NC). After 48 h of co-transfection, cell lysates were obtained using Passive Lysis Buffer (Promega, USA) . Dual Luciferase Assay Kit (Promega) and GloMax 20/20 Luminometer (Promega) were used to examine the relative luciferase activity. The relative luciferase activity in each sample was normalized by the ratio of firefly/Renilla luciferase activity.

Immunofluorescence

HCC cells were cultured on coverslips in 24-well plates for 24 h. After 3 washes with phosphate-buffered saline, the cells were fixed in 4% paraformaldehyde for 30 min and permeabilized with 0.1% Triton X-100 for 10 min. After culturing in medium containing 1% bovine serum albumin for 2 h, the cells were incubated with primary antibody overnight at 4°C and then labeled with appropriate secondary antibody at the room temperature for 2 h. Cell nuclei were stained with 4′,6-diamidino-2-phenylindole for 10 min. The coverslips were mounted and observed with confocal laser scanning microscopy (Leica, Germany).

The organoid culture medium

The organoid culture medium contained: Advanced DMEM/F12 supplemented with 1% penicillin/streptomycin, 1% glutamax, 10-mM HEPES, 1:50 B27 supplement (without vitamin A), 1:100 N2 supplement, 1.25-mM N-acetyl-l-cysteine, 10-mM nicotinamide, 50 ng/ml recombinant human EGF, 50 ng/ml recombinant human FGF-10, 25 png/ml recombinant human HGF, 500ng/mL R-spondin1, 100ng/mL Wnt-3a, 50ng/mL TGFa, 5uM forskolin, 5 uM A8301, 10 μM Y27632.

RNA-seq data analysis

RNA-seq, GO and KEGG enrichment analysis were conducted by Haplox (Jiang Xi, China)

References

1. Hou R, Wang YW, Liang HF, et al. Animal and cellular models of hepatocellular carcinoma bone metastasis: establishment and characterisation. *J Cancer Res Clin Oncol* 2015; 141(11):1931-43.
